# Measurement and Interpretation of Exercise Ventilatory Efficiency

**DOI:** 10.3389/fphys.2020.00659

**Published:** 2020-06-25

**Authors:** Devin B. Phillips, Sophie É. Collins, Michael K. Stickland

**Affiliations:** ^1^ Clinical Physiology Laboratory, Division of Pulmonary Medicine, Faculty of Medicine & Dentistry, University of Alberta, Edmonton, AB, Canada; ^2^ Faculty of Kinesiology, Sport, and Recreation, University of Alberta, Edmonton, AB, Canada; ^3^ Faculty of Rehabilitation Medicine, University of Alberta, Edmonton, AB, Canada; ^4^ G.F. MacDonald Centre for Lung Health, Covenant Health, Edmonton, AB, Canada

**Keywords:** ventilatory efficiency, ventilation, exercise testing, dyspnea, pulmonary gas-exchange

## Abstract

Cardiopulmonary exercise testing (CPET) is a method for evaluating pulmonary and cardiocirculatory abnormalities, dyspnea, and exercise tolerance in healthy individuals and patients with chronic conditions. During exercise, ventilation (*V˙*_E_) increases in proportion to metabolic demand [i.e., carbon dioxide production (*V˙*CO_2_)] to maintain arterial blood gas and acid-base balance. The response of *V˙*_E_ relative to *V˙*CO_2_ (*V˙*_E_/*V˙*CO_2_) is commonly termed ventilatory efficiency and is becoming a common physiological tool, in conjunction with other key variables such as operating lung volumes, to evaluate exercise responses in patients with chronic conditions. A growing body of research has shown that the *V˙*_E_/*V˙*CO_2_ response to exercise is elevated in conditions such as chronic heart failure (CHF), pulmonary hypertension (PH), interstitial lung disease (ILD), and chronic obstructive pulmonary disease (COPD). Importantly, this potentiated *V˙*_E_/*V˙*CO_2_ response contributes to dyspnea and exercise intolerance. The clinical significance of ventilatory inefficiency is demonstrated by findings showing that the elevated *V˙*_E_/*V˙*CO_2_ response to exercise is an independent predictor of mortality in patients with CHF, PH, and COPD. In this article, the underlying physiology, measurement, and interpretation of exercise ventilatory efficiency during CPET are reviewed. Additionally, exercise ventilatory efficiency in varying disease states is briefly discussed.

## Introduction

Cardiopulmonary exercise testing (CPET) is used for clinical evaluation of pulmonary and cardiocirculatory abnormalities, breathlessness (termed dyspnea), and exercise tolerance in patients with chronic conditions (Palange et al., 2007). CPET is a helpful diagnostic tool that can aid clinicians to identify patterns of functional impairment that may not be recognized by resting physiological testing (i.e., pulmonary function test, echocardiogram) and is particularly well suited to understand factors that may lead to pulmonary-related limitations to exercise (Stickland et al., 2012). Further, emerging research suggests that resting physiological tests poorly predict exertional dyspnea and exercise intolerance in clinical populations and that CPET is vital to evaluate causes of previously unexplained dyspnea and exercise intolerance (Elbehairy et al., 2015, 2017; Neder et al., 2016; Boucly et al., 2020).

During exercise, ventilation (*V˙*_E_) increases in proportion to metabolic demand [i.e., carbon dioxide production (*V˙*CO_2_)] in order to maintain acid-base balance. The response of *V˙*_E_ relative to *V˙*CO_2_ (*V˙*_E_/*V˙*CO_2_), said to reflect ventilatory efficiency (Forster and Pan, 1988), has become a common physiological tool, in conjunction with other key variables such as operating lung volumes and subjective dyspnea ratings, to evaluate exercise responses in patients with chronic conditions. For clinicians to incorporate ventilatory efficiency into CPET interpretation, a thorough understanding of the background physiology of ventilatory efficiency is needed. This article provides a detailed review on the underlying physiology, measurement, and interpretation of exercise ventilatory efficiency during CPET. Additionally, exercise ventilatory efficiency in varying disease conditions is briefly discussed.

### Ventilatory Responses During Exercise

Alveolar ventilation (*V˙*_A_) represents the ventilation which takes part in gas-exchange. The relationship between *V˙*_A_, CO_2_ production (*V˙*CO_2_), and the partial pressure of alveolar CO_2_ (P_A_CO_2_) is defined by

(1) $$P_{A}{\mathrm{CO}}_{2}={\left(V˙{\mathrm{CO}}_{2}/V˙\right)}_{A}\times K\;$$

where *K* is a conversion factor (normally = 863) used to adjust *V˙*CO_2_ from standard temperature and pressure dry (STPD) to body temperature, ambient pressure, saturated (BTPS). As demonstrated by equation (1), P_A_CO_2_ is determined by the relative balance of *V˙*CO_2_ and *V˙*_A_. Due to the difficulty in measuring P_A_CO_2_, arterial PCO_2_ (P_a_CO_2_) is often used as a surrogate with the assumption that P_A_CO_2_ ≈ P_a_CO_2_ (Enghoff, 1938). Well-matched alveolar ventilation to perfusion is critical to maximize ventilatory efficiency and minimize dead space ventilation (West and Dollery, 1960; West et al., 1964). Although the healthy lung generally has good matching of ventilation to perfusion (West and Dollery, 1960; Whipp and Ward, 1982), a portion of gas remains in the conducting airways and does not participate in gas-exchange, and is termed anatomical dead space. Alveolar dead space represents the fraction of alveoli that are ventilated but not perfused. Dead space ventilation is the sum of alveolar and anatomical dead space, and, thus total minute ventilation is the sum of alveolar and dead space ventilation, as displayed in equation (2):

(2) $$V{˙}_{E}=V{˙}_{A}+V{˙}_{D}$$

Minute ventilation is measured at the mouth with expired gas analysis; however, *V˙*_A_ and *V˙*_D_ are more difficult to determine. If arterial blood gas and expired gas data are available, it is possible to derive *V˙*_A_ from equation (1), or estimate total physiologic dead space as a proportion of tidal volume using Enghoff’s modified Bohr equation (Enghoff, 1938):

(3) $$V_{D}/V_{T}=\left(P_{a}{\mathrm{CO}}_{2}-P_{E}{\mathrm{CO}}_{2}\right)/\left(P_{a}{\mathrm{CO}}_{2}\right)$$

In equation (3), P_E_CO_2_ represents mixed expired partial pressures of CO_2_ and *V*_T_ is tidal volume. It is often assumed that the end-tidal partial pressure of CO_2_ (P_ET_CO_2_) can be used as a surrogate for P_a_CO_2_. However, previous research has shown discrepancies between arterial blood-gas derived P_a_CO_2_ and expired-gas derived P_ET_CO_2_ in patients with pulmonary gas-exchange abnormalities (Scheidl et al., 2012; Laveneziana et al., 2014b; Elbehairy et al., 2015). Ventilation-perfusion (*V˙*_A_/*Q˙*) abnormalities (typically alveolar dead space) results in dilution of gas from poorly perfused alveoli, which lowers the end-tidal CO_2_ concentration. As a result, P_ET_CO_2_ often underestimates P_a_CO_2_ in patients with increased alveolar dead space such as pulmonary hypertension (PH) and chronic obstructive pulmonary disease (COPD; Scheidl et al., 2012; Laveneziana et al., 2014b; Elbehairy et al., 2015). Because of the potential error in determining P_a_CO_2_ from end-tidal data, care should be given when interpreting dead space calculated only with end-tidal and mixed expired values.

**Figure 1:**
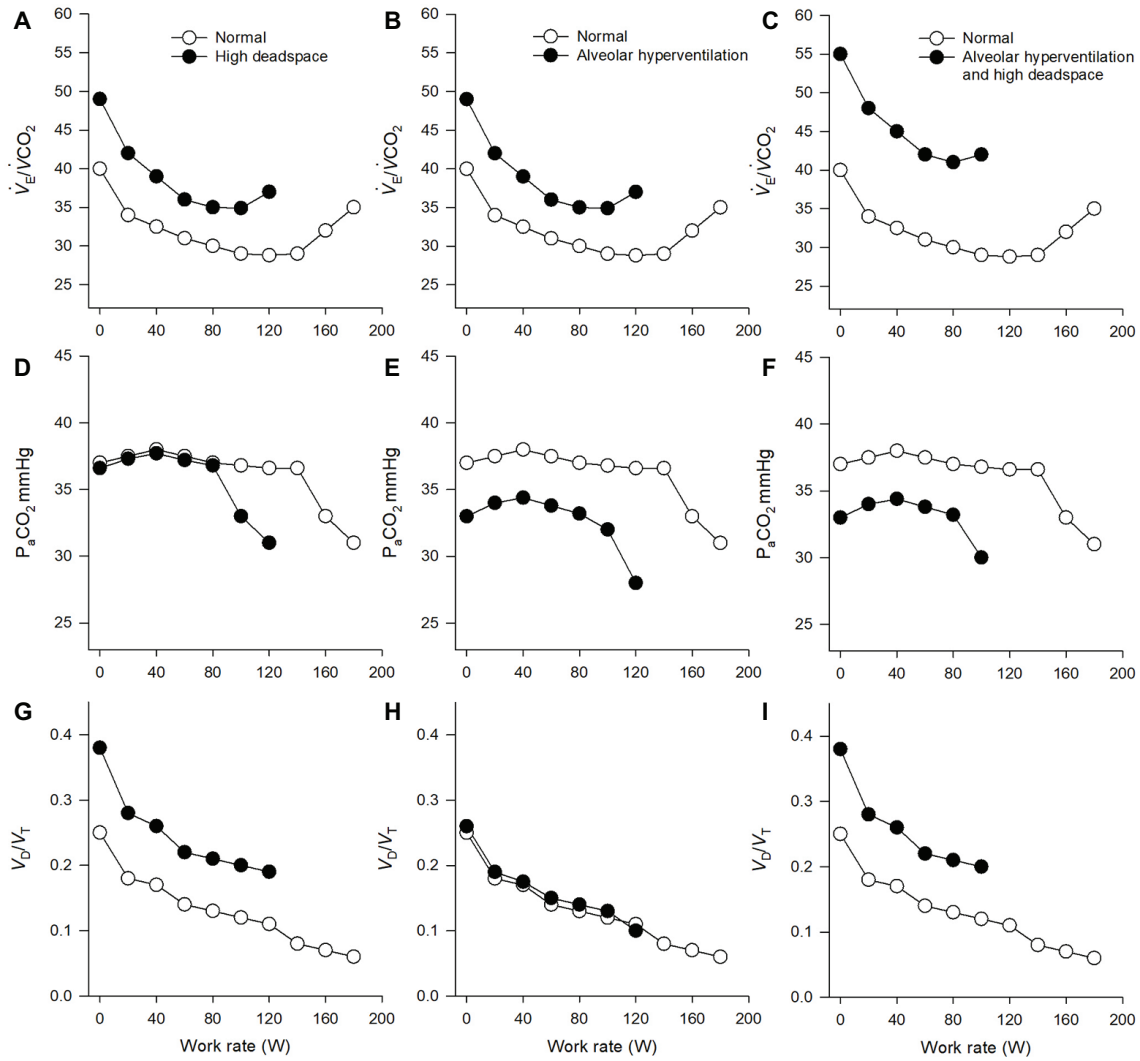
Ventilatory and gas-exchange responses to incremental exercise. The left columns **(A,D,G)** display theoretical normal responses (open circles) and abnormal responses (closed circles). The theoretical abnormal responses demonstrate an elevated ventilatory response to carbon dioxide output (*V˙*_E_/*V˙*CO_2_), secondary to elevated dead space (dead space to tidal volume ratio = *V*_D_/*V*_T_) and normal partial pressure of arterial carbon dioxide (P_a_CO_2_). The middle columns **(B,E,H)** display theoretical normal responses (open circles) and abnormal responses (closed circles). The theoretical abnormal responses demonstrate an elevated *V˙*_E_/*V˙*CO_2_, secondary to alveolar hyperventilation (reduced P_a_CO_2_) but relatively normal dead space. The right columns **(C,F,I)** display theoretical normal responses (open circles) and abnormal responses (closed circles). The theoretical abnormal responses demonstrate an elevated *V˙*_E_/*V˙*CO_2_, secondary to a combination of alveolar hyperventilation (reduced P_a_CO_2_) and elevated dead space.

Based on the equations outlined above, the total ventilatory requirement to remove metabolic CO_2_ production (*V˙*_E_/*V˙*CO_2_) is elevated in the presence of alveolar hyperventilation and/or high dead space. In conditions of hyperventilation assuming normal dead space, *V˙*_E_/*V˙*CO_2_ would be elevated and P_a_CO_2_ lowered (Figure 1). As discussed below, many clinical conditions demonstrate hyperventilation during exercise, secondary to increased afferent feedback (including but not limited to skeletal muscle ergoreceptors, chemoreceptors, and baroreceptors) (Ponikowski et al., 1997, 2001; Scott et al., 2000; Farina et al., 2018). In conditions of increased total physiologic dead space (i.e., high *V*_D_/*V*_T_) during exercise, *V˙*_E_ must increase to maintain adequate *V˙*_A_ and blood gas homeostasis (i.e., P_a_CO_2_; Figure 1). In addition to classic alveolar dead space (i.e., ventilation with no perfusion), *V*_D_/*V*_T_ will be elevated when patients adopt a shallow, tachypneic breathing response. Hyperventilation also results in a rightward shift in the overall *V˙*_A_/*Q˙* relationship which can directly increase *V*_D_/*V*_T_ (West, 1969; Robertson, 2015). In many chronic diseases, both elevated dead space and alveolar hyperventilation coexist (Figure 1) and, without measurement of P_a_CO_2_, it becomes difficult to quantify the contribution of each.

### Clinical Significance of Ventilatory Efficiency

It is accepted that perceived dyspnea increases as ventilatory demand rises during incremental exercise, even in healthy individuals (Ofir et al., 2008a,b; Faisal et al., 2015). Ventilatory inefficiency increases the ventilatory demand for a given metabolic load which results in: (1) earlier attainment of critical dynamic mechanical constraint (i.e., critically reduced inspiratory reserve volume) and (2) increased respiratory neural drive, both of which lead to heightened perceived dyspnea and exercise intolerance, even in individuals with relatively preserved airway function (Guenette et al., 2014; Faisal et al., 2016). Multiple studies have linked ventilatory inefficiency to abnormal exertional dyspnea and poor exercise capacity in varying cardiocirculatory and respiratory diseases (Hansen and Wasserman, 1996; Arena et al., 2004; Neder et al., 2015, 2016; Elbehairy et al., 2017; Ewert et al., 2019). Additionally, the elevated *V˙*_E_/*V˙*CO_2_ response to exercise is an independent predictor of poor outcomes and mortality in many diseases, including chronic heart failure (CHF), PH, and COPD (Ponikowski et al., 2001; Wensel et al., 2013; Neder et al., 2016). Thus, the clinical utility of assessing ventilatory efficiency during CPET may be helpful to explain reasons for exertional dyspnea and exercise intolerance in various underlying disease states.

## Measuring and Interpreting Ventilatory Efficiency From Cpet

*V˙*_E_/*V˙*CO_2_ is easily included in a standard CPET report; however, careful consideration must be taken when analyzing and interpreting the data. This section describes common methods of reporting ventilatory efficiency from CPET data and technical considerations that may influence interpretation.

### *V˙*_E_-to-*V˙*CO_2_ Slope and *y*-Intercept

**Figure 2:**
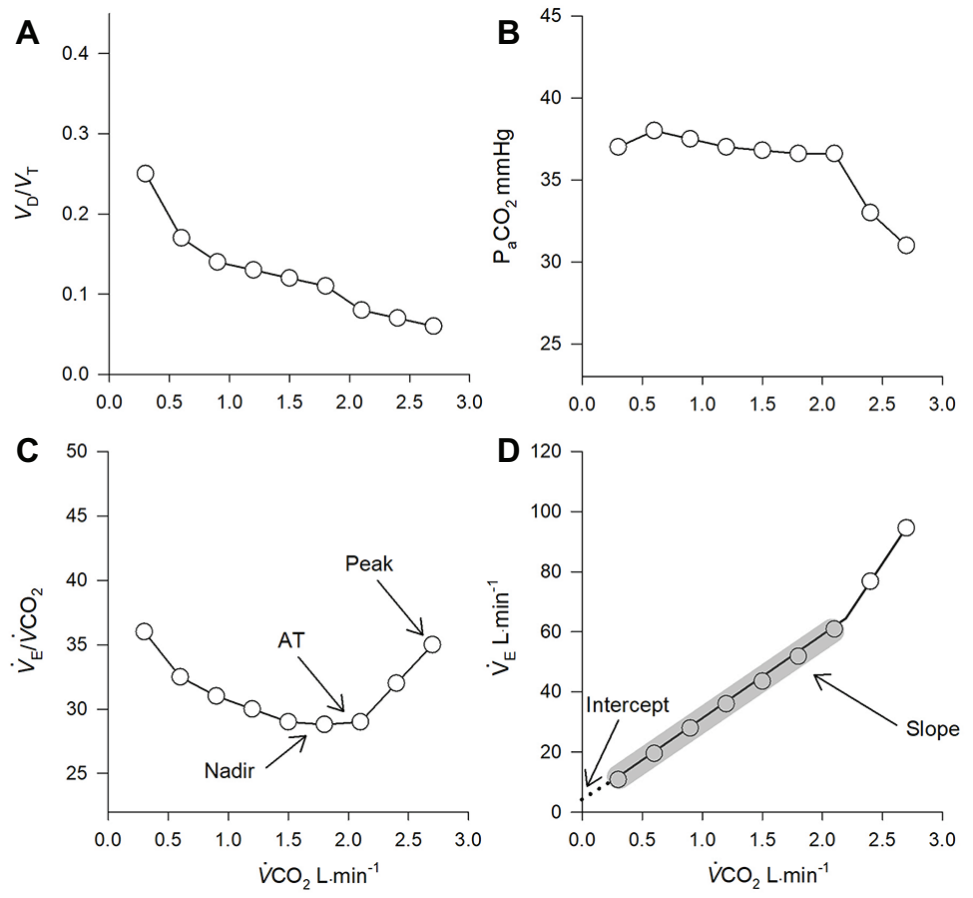
Theoretical gas-exchange **(A, B)** and ventilatory **(C)** responses to incremental exercise. **(D)** represents the ventilatory responses to increasing metabolic demand. P_a_CO_2_ = partial pressure of arterial carbon dioxide; *V*_D_/*V*_T_ = deadspace to tidal volume ratio; *V˙*_E_/*V˙*CO_2_ = ventilatory equivalent for carbon dioxide; AT = anaerobic threshold. Data points within grey shading represent the values used to calculate the slope of the regression line (note, the final two data points were excluded in the calculation as they occurred after the respiratory compensation point). The dashed line in **(D)** represents linear interpolation to the *y*-intercept.

During incremental exercise to symptom limitation, the relationship between *V˙*_E_ and *V˙*CO_2_ can be reported by plotting *V˙*_E_ (*y*-axis) relative to *V˙*CO_2_ (*x*-axis, Figure 2). The *V˙*_E_-to-*V˙*CO_2_ relationship during exercise can then be determined by analyzing the slope of this regression line (Whipp and Ward, 1982). Previous research has shown that the *V˙*_E_-to-*V˙*CO_2_ slope lower and upper limits of normal range from approximately 21 to 31 units, respectively (Sun et al., 2002; Naeije and Faoro, 2018). The *V˙*_E_-to-*V˙*CO_2_ slope is considered one of the most robust indicators of ventilatory efficiency, assuming ventilatory responses are not impaired by abnormal ventilatory mechanics (Neder et al., 2017).

During light to heavy exercise, *V˙*_E_ changes as a linear function of *V˙*CO_2_. Importantly, at heavy exercise above the respiratory compensation point, *V˙*_E_ rises disproportionally to *V˙*CO_2_ due to excessive metabolic acidosis. In individuals who tolerate high levels of exercise, an upward inflection in the *V˙*_E_ relative to *V˙*CO_2_ response would occur at maximal exercise and would inflate the *V˙*_E_-to-*V˙*CO_2_ slope if all data points are included in analysis. In these cases, determination of the slope of the regression line should exclude the non-linear portion (i.e., data after the respiratory compensation point should be excluded) (Arena et al., 2003; Guazzi et al., 2005).

When analyzing the *V˙*_E_-to-*V˙*CO_2_ slope in respiratory disease, such as COPD, the interpretation becomes more complex. Respiratory mechanical constraint and airflow limitation are often observed in these patients, which can blunt the rise in ventilation during exercise. As such, the *V˙*_E_-to-*V˙*CO_2_ slope paradoxically decreases as COPD severity worsens (Neder et al., 2015) (see section COPD Chronic Obstructive Pulmonary Disease). Thus, clinicians must look beyond the *V˙*_E_-to-*V˙*CO_2_ slope when evaluating ventilatory efficiency from CPET, especially in patients with respiratory mechanical constraints.

In addition to the slope, the *y*-intercept of the *V˙*_E_-to-*V˙*CO_2_ relationship (i.e., *V˙*_E_ when *V˙*CO_2_ = 0) can be determined from the same regression analysis (Figure 2). An elevated *y*-intercept is indicative of an upward shift in *V˙*_E_ for a given *V˙*CO_2_ and is considered to be an index of ventilatory efficiency at rest and during light exercise (i.e., at the start of a CPET) (Neder et al., 2015). The *y*-intercept can be a useful tool in the event of a premature test termination, as a maximal effort is not required. Recent research has demonstrated the *y*-intercept may help differentiate CPET patterns between COPD and CHF with overlapping symptoms (i.e., dyspnea and exercise intolerance), as COPD patients consistently demonstrate an elevated *y*-intercept, compared to CHF (Smith et al., 2019).

### *V˙*_E_/*V˙*CO_2_ Nadir

Generally, in the transition from light to moderate intensity exercise, P_a_CO_2_ remains constant or slightly increases, while V_D_/V_T_ decreases. As a result, the *V˙*_E_/*V˙*CO_2_ ratio is elevated during light exercise at the start of a CPET and progressively decreases in tandem with *V*_D_/*V*_T_ to its lowest value (nadir) just prior to the respiratory compensation point (Figure 2; Whipp and Ward, 1982). The nadir *V˙*_E_/*V˙*CO_2_ is often considered the most accurate assessment of ventilatory efficiency, as it occurs independent of (1) the excess *V˙*_E_/*V˙*CO_2_ response to low intensity exercise and (2) metabolic acidosis and respiratory compensation during heavy exercise (Whipp and Ward, 1982). In healthy individuals, the *V˙*_E_/*V˙*CO_2_ corresponding to the nadir and the *V˙*_E_/*V˙*CO_2_ at anaerobic threshold are often similar (Figure 2; Sun et al., 2002). The nadir *V˙*_E_/*V˙*CO_2_ increases progressively with age and is abnormally high in cardiocirculatory and respiratory disease (Sun et al., 2002; Ingle et al., 2012; Elbehairy et al., 2015, 2019; Neder et al., 2015; Phillips et al., 2019). Although the nadir *V˙*_E_/*V˙*CO_2_ is highly reproducible, it may over-estimate ventilatory inefficiency in individuals with poor exercise tolerance and an excessively short test duration during CPET (Neder et al., 2001).

### *V˙*_E_/*V˙*CO_2_ at Peak Exercise

At peak exercise, *V˙*_E_/*V˙*CO_2_ is often much higher than the nadir value as individuals typically hyperventilate secondarily to excessive metabolic acidosis (Figure 2). However, in individuals with poor exercise tolerance who are generally unable to exercise above anaerobic threshold, the nadir and peak exercise *V˙*_E_/*V˙*CO_2_ values are often similar (Neder et al., 2015; Phillips et al., 2019). Peak exercise *V˙*_E_/*V˙*CO_2_ is considered a poor index of ventilatory efficiency in healthy individuals and in patients who can exercise above the anaerobic threshold (Neder et al., 2017). For these reasons, clinicians should use caution when using peak exercise *V˙*_E_/*V˙*CO_2_ values as an index of ventilatory efficiency.

## Ventilatory Efficiency in Various Conditions

### Healthy Aging

Healthy aging is associated with an increased *V˙*_E_/*V˙*CO_2_ (slope and nadir) response to exercise, when compared to younger healthy individuals (Sun et al., 2002; Faisal et al., 2015). The elevated *V˙*_E_/*V˙*CO_2_ response is likely due to increased dead space, secondary to age-related reductions in alveolar-capillary surface area, capillary blood volume, and *V˙*_A_/*Q˙* mismatch (Tenney and Miller, 1956; Raine and Bishop, 1963; Johnson and Dempsey, 1991; Cardus et al., 1997). However, age-related ventilatory inefficiency is generally not a primary cause of exercise limitation in older healthy individuals.

### Chronic Heart Failure

Multiple studies have demonstrated that patients with CHF with reduced ejection fraction (HFrEF) have an elevated ventilatory response to exercise, which is a key contributor to exertional dyspnea and exercise intolerance. The elevated *V˙*_E_/*V˙*CO_2_ (slope and nadir) is generally ascribed to hyperventilation, secondary to increased afferent feedback from chemoreceptors, baroreceptors, and ergoreceptors (Ponikowski et al., 1997, 2001; Scott et al., 2000). CHF patients typically adopt a rapid-shallow breathing pattern, which would increase the *V*_D_/*V*_T_ ratio and contribute to a greater *V˙*_E_/*V˙*CO_2_ (Woods et al., 2010). As described above, part of the increased *V*_D_/*V*_T_ in CHF is likely due to the hyperventilation itself, which results in a rightward shift of a normal *V˙*_A_/*Q˙* distribution to a higher mean *V˙*_A_/*Q˙* ratio, and greater dead space (Robertson, 2015). The contribution of both hyperventilation (low P_a_CO_2_) and elevated dead space contributing to an exaggerated *V˙*_E_/*V˙*CO_2_ is demonstrated by Johnson (2001) who further analyzed individual CHF exercise data from Franciosa et al. (1984). A low peak P_a_CO_2_ was associated with a high peak *V˙*_E_/*V˙*CO_2_. Of note, a low cardiac index was also associated with a high *V˙*_E_/*V˙*CO_2_ (suggesting those with a low cardiac output likely have a higher mean *V˙*_A_/*Q˙* ratio and thus greater dead space). Finally, a high *V*_D_/*V*_T_ ratio was correlated with a high *V˙*_E_/*V˙*CO_2_ (Franciosa et al., 1984; Johnson, 2001). When combined, these data suggest both hyperventilation and increased dead space contribute to the increased *V˙*_E_/*V˙*CO_2_ during exercise in CHF.

Emerging work has demonstrated ventilatory inefficiency (elevated *V˙*_E_-to-*V˙*CO_2_ slope) in patients with CHF with preserved ejection fraction (HFpEF; Olson et al., 2016); however, the mechanism(s) for the elevated *V˙*_E_/*V˙*CO_2_ is unclear. Patients with HFpEF often have co-existing PH (Lam et al., 2009), which may help explain the elevated *V˙*_E_-to-*V˙*CO_2_ slope; however, future work is required to better understand the pulmonary gas-exchange abnormalities in this population.

### Pulmonary Hypertension

PH is characterized by a heightened ventilatory response to exercise (i.e., elevated *V˙*_E_-to-*V˙*CO_2_ slope) (Sun et al., 2001; Zhai et al., 2011). Patients with PH demonstrate significant ventilation-perfusion abnormalities, which is evident by an increased mean *V˙*_A_/*Q˙* ratio, larger than normal arterial-end-tidal PCO_2_ differences, and increased dead space (*V*_D_/*V*_T_), all of which contribute to the elevated *V˙*_E_/*V˙*CO_2_ response during exercise (Dantzker et al., 1984; Zhai et al., 2011; Laveneziana et al., 2013). Although elevated dead space is generally considered the primary reason for ventilatory inefficiency in PH, previous work has shown that these patients hyperventilate at rest (as demonstrated by a low P_a_CO_2_) and demonstrate significant hyperventilation during exercise (Laveneziana et al., 2013, 2014a; Farina et al., 2018). Like CHF, patients with PH often have a blunted cardiac output response to exercise. The combination of low cardiac output and hyperventilation would cause a similar upward shift in the mean *V˙*_A_/*Q˙* ratio as observed in CHF, which may partially explain the elevated dead space typically observed in PH (Dantzker et al., 1984). When combined, it is evident that the increased *V˙*_E_/*V˙*CO_2_ response to exercise in PH is explained by both hyperventilation and increased dead space. Readers are referred to a review by Robertson (2015) that provides an excellent summary of current understanding of factors contributing to exercise hyperventilation in patients with cardiocirculatory disease.

### Chronic Obstructive Pulmonary Disease

Airflow limitation, secondary to dynamic respiratory mechanical abnormalities, is generally considered the primary cause of exertional dyspnea in COPD. However, emerging research has suggested that ventilatory inefficiency is also a key contributor to dyspnea and exercise intolerance in patients with COPD (Neder et al., 2015; Elbehairy et al., 2019). In a study examining patients with mild COPD, arterial blood gas-derived dead space and *V˙*_E_/*V˙*CO_2_ (slope, nadir, and *y*-intercept) were consistently elevated during exercise while P_a_CO_2_ and alveolar ventilation were similar to age-matched healthy controls (Elbehairy et al., 2015). These data suggest that ventilatory inefficiency during exercise in mild COPD is primarily due to elevated dead space.

With advancing COPD (moderate to very severe), the *V˙*_E_/*V˙*CO_2_ response to exercise is more complex. With increasing COPD severity, dead space increases and is markedly elevated in the presence of extensive emphysematous destruction of capillary beds (Rodriguez-Roisin et al., 2009). In these patients, the elevated anatomical and total dead space is further amplified by the adopted rapid and shallow breathing pattern, secondary to severe hyperinflation, critically low inspiratory reserve volume, and limited tidal volume expansion (O᾽Donnell et al., 2012). Additionally, patients with COPD often become hypoxemic (Stolz et al., 2014), which would stimulate peripheral chemoreceptors and further increase ventilatory drive. The pronounced dead space and reduced P_a_O_2_ combined with a rapid shallow breathing would result in a substantially elevated *V˙*_E_/*V˙*CO_2_ response at rest and during light exercise; however, the pronounced airflow limitation in these patients often results in an inability to increase ventilation appropriately at higher metabolic demands. The net result is that patients with moderate to very severe COPD typically demonstrate an increased *V˙*_E_-to-*V˙*CO_2_ *y*-intercept and nadir *V˙*_E_/*V˙*CO_2_ but low *V˙*_E_-to-*V˙*CO_2_ slope and *V˙*_E_/*V˙*CO_2_ at peak exercise (Neder et al., 2015).

### Interstitial Lung Disease

A growing body of research has shown that pulmonary gas-exchange abnormalities and ventilatory inefficiency (i.e., elevated *V˙*_E_/*V˙*CO_2_ slope and nadir) are key mechanisms of dyspnea and exercise intolerance in patients with interstitial lung disease (ILD; Faisal et al., 2016; Schaeffer et al., 2018). The increased *V˙*_E_/*V˙*CO_2_ during exercise in ILD appears to be due to the combination of increased dead space and hyperventilation (Agusti et al., 1991; Faisal et al., 2016). During exercise in patients with ILD, dynamic restrictive mechanical constraints, secondary to increased lung elastic recoil, increase inspiratory elastic loading, and patients often adopt a more rapid and shallow breathing pattern during exercise to minimize the inspiratory elastic work of breathing (Faisal et al., 2016; Schaeffer et al., 2018). Although the reduction in tidal volume is an effective strategy to minimize the elastic work of breathing during exercise, the compensatory tachypnea increases anatomical dead space and, ultimately, *V˙*_E_/*V˙*CO_2_ (Faisal et al., 2016; Schaeffer et al., 2018).

Multiple studies have consistently shown that patients with ILD develop exercise-induced arterial hypoxemia, which would stimulate the peripheral chemoreceptors and increase ventilatory drive, ultimately increasing *V˙*_E_/*V˙*CO_2_ (Hamer, 1964; Agusti et al., 1991; Faisal et al., 2016). Patients with ILD, specifically idiopathic pulmonary fibrosis, often have PH which may worsen pulmonary gas-exchange and exacerbate the already elevated ventilatory response to exercise (Lettieri et al., 2006; Nathan et al., 2007); however, future work is required to better understand how abnormal pulmonary hemodynamics affects pulmonary gas-exchange in this population.

## Conclusions

Measurement of ventilatory efficiency, as determined by the *V˙*_E_/*V˙*CO_2_ response to exercise, is an important physiological component, in conjunction with other key variables, to evaluate exercise responses and help explain reasons for exertional dyspnea in different underlying disease states. While measurements of ventilatory efficiency can aid clinicians to interpret CPET results, it is important to understand the strengths and limitations of the various methods of interpreting ventilatory efficiency data and understand the underlying pathophysiology.

## Author Contributions

All authors contributed to the article and approved the submitted version.

## Conflict of Interest

The authors declare that the research was conducted in the absence of any commercial or financial relationships that could be construed as a potential conflict of interest.
